# Development of a luciferase/luciferin cell proliferation (XenoLuc) assay for real-time measurements of Gfp-Luc2-modified cells in a co-culture system

**DOI:** 10.1186/s12896-019-0528-4

**Published:** 2019-06-14

**Authors:** Sin-Yeang Teow, Kitson Liew, Mohd Firdaus Che Mat, Marini Marzuki, Norazlin Abdul Aziz, Tai-Lin Chu, Munirah Ahmad, Alan Soo-Beng Khoo

**Affiliations:** 1 Molecular Pathology Unit, Cancer Research Centre, Institute for Medical Research, National Institutes of Health (NIH Complex), Ministry of Health Malaysia, Level 4, Block C7, No: 1, Jalan Setia Murni U13/52, Section U13, Setia Alam, 40170 Shah Alam, 50588 Kuala Lumpur, Selangor Malaysia; 2 Present Address: Department of Medical Sciences, School of Healthcare and Medical Sciences, Sunway University, 47500 Bandar Sunway, Selangor Darul Ehsan Malaysia

**Keywords:** Luciferase assay, Luminescence, Nasopharyngeal carcinoma, Nasopharyngeal neoplasms, Patient-derived xenograft, Fibroblasts, Tumour microenvironment, Stromal interaction, Proliferation, Co-culture, Spheroids, Drug screening, Cancer immunology

## Abstract

**Background:**

In vitro modelling of cancer cells is becoming more complex due to prevailing evidence of intimate interactions between cancer cells and their surrounding stroma. A co-culture system which consists of more than one cell type is physiologically more relevant and thus, could serve as a useful model for various biological studies. An assay that specifically detects the phenotypic changes of cancer cells in a multi-cellular system is lacking for nasopharyngeal carcinoma (NPC).

**Results:**

Here, we describe a luciferase/luciferin (XenoLuc) assay that could specifically measure changes in the proliferation of cancer cells in the co-culture system using two modified NPC patient-derived tumour xenograft (PDTXs) cells: Xeno284-gfp-luc2 and XenoB110-gfp-luc2. Through this assay, we are able to show that the growth of NPC xenograft cells in both two-dimensional (2D) and three-dimensional (3D) models was enhanced when co-cultured with normal human dermal fibroblasts (NHDFs). In addition, potential applications of this assay in in vitro drug or inhibitor screening experiments are also illustrated.

**Conclusions:**

XenoLuc assay is specific, sensitive, rapid and cost-effective for measuring the growth of luciferase-expressing cells in a co- or multiple-culture system. This assay may also be adapted for tumour microenvironment studies as well as drug screening experiments in more complex 3D co-culture systems.

**Electronic supplementary material:**

The online version of this article (10.1186/s12896-019-0528-4) contains supplementary material, which is available to authorized users.

## Background

Cell-based assays remain the leading platform for cancer research in preclinical settings. Majority of high-throughput compound screening programmes carried out by major pharmaceutical companies utilise cell-based assays to identify high-quality leads [1]. Such assays have the advantage over biochemical-based assays since these assays provide information on the cellular responses of a particular drug candidate. Moreover, cell-based assays could be reliably used to provide early indications of drug toxicity [1]. The recent advancement in cell culture technology has paved way for the development of in vitro cell-based assays that could be carried out in a more biologically relevant microenvironments. Such assays may include the incorporation of extracellular matrices (ECMs) and scaffolds as well as stromal cells in the presence of tumour cells [2, 3]. However, challenges remain in the design of these assays, particularly on measuring phenotypic changes of a specific cell population in complex multi-cell type systems. Notably, there is a scarcity of convenient cell-based assays that could discriminate phenotypes of different cell types in a co-culture setting [4, 5]. With greater understanding of the microenvironment surrounding a tumour, it is increasingly important to employ appropriate in vitro assays that are able to address issues of tumour complexity and heterogeneity [2].

Cancer cells are often grown as monolayers, of which 2D (monolayer) culture models are commonly employed for in vitro drug testing. However, these 2D models are bound by several limitations that may potentially negate their usefulness. For example, there is a lack of 3D cell-cell and cell-ECM interactions in 2D models that are present in in vivo models [6]. These interactions generate signalling cues that are pivotal for numerous important cellular functions such as proliferation, differentiation and survival. Most of these functions are lost or have been compromised in 2D culture models. As a result, data generated from 2D in vitro drug testing could be misleading and non-predictive for in vivo responses [6]. The spatial arrangement of cancer cells within the 3D model, possibly together with the presence of other cell types and ECM components, mimic their natural microenvironment and hence, may reinstate the important signalling that are lost or compromised in 2D culture systems [7]. For these reasons, 3D culture models offer greater clinical and biological relevance than in vitro models and therefore, could bridge the gap between the monolayer culture and animal works.

Our group had developed two PDTXs, XenoB110 and Xeno284 from Malaysian NPC patients [8]. To facilitate the monitoring of in vivo tumour growth, these PDTXs were co-transduced with gfp-luc2 reporter genes (XenoB110-gfp-luc2 and Xeno284-gfp-luc2). The XenoLuc assay described in this paper is a luciferase-based assay that uses commercial D-Luciferin as the substrate for the luciferase in the transduced PDTXs to generate a luminescent signal that is proportional to cell numbers. We demonstrate that the XenoLuc assay is sensitive and could specifically measure real-time proliferation of PDTXs both in vitro and in vivo. As 3D models are gaining prominence as in vitro cell-based assays, we also demonstrate the robustness of XenoLuc assay in both mono- and multi-cellular spheroid cultures in addition to the conventional 2D monolayer model. This assay is able to measure the PDTXs growth enhancement that resulted from the addition of growth supplements as well as from the effect of co-culturing with other human cell types. In addition, the assay may also be used to evaluate the loss of cell viability, such as that induced by cisplatin treatment. Since the proliferation and viability of PDTX cells could be specifically detected in the complex 3D co-culture systems, XenoLuc assay may represent a cost-effective and a potentially useful cell-based assay for studies on tumour microenvironment, high-throughput compound screening and preclinical drug response prediction.

## Results

### Specificity and sensitivity of XenoLuc assay

**Fig. 1:**
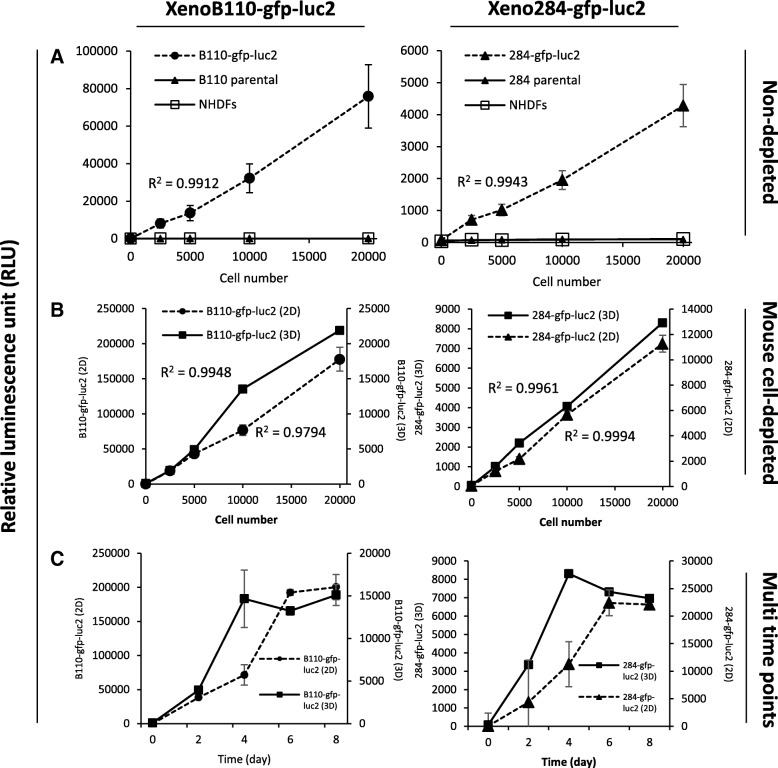
XenoLuc assay for in vitro cell proliferation measurement. **a** Measurement of luminescence in two-fold serially diluted non-depleted xenograft cells (parental and modified) and NHDF cells. These cells were seeded onto 96-well plates as 2D monolayer culture, and the luminescence was measured after 4 days. **b** Measurement of luminescence of mouse cell-depleted xenograft cells cultured in vitro in 2D and 3D models. **c** Proliferation rate of depleted xenografts cells as measured by XenoLuc assay at a 2-day interval. Results are represented by average of triplicate from 3 mice ± SD. Left panel: XenoB110-gfp-luc2; Right panel: Xeno284-gfp-luc2. R^2^ shows the linear correlation of luminescence and cell number

The specificity of XenoLuc assay was assessed by determining if it specifically detected luminescent signals from luciferase-expressing cells compared to non-expressing cells. All experiments were carried out using freshly isolated xenograft cells harvested from three separate tumours from different mice and used for not more than two passages. Non-treated/non-cultured cells are used in assays as internal controls to avoid batch-to-batch variation which could arise from multiple causes. Based on Fig. 1a, luminescence was detected in *luc2*-transduced xenograft cells but not in parental xenograft cells and NHDFs, indicating the specificity of XenoLuc assay. Additional file 1: Figure S1 shows the linear correlation between luminescence signals versus various titrations of XenoB110-gfp-luc2 and Xeno284-gfp-luc2 cells measured at day-0 (30 min after cell seeding when the cells had settled at the bottom of wells, but had not adhered). The mouse cell-depleted xenograft cells exhibited higher luminescent signals compared to the non-depleted xenograft cells (Fig. 1a and b). To examine the assay sensitivity, two-fold serial dilutions of cells were plated and the readings were taken after 4 days. Figure 1b shows that cell seeding number had a positive linear correlation with luminescence in both 2D and 3D culture models, of which the lowest seeding density tested was 2500 cells/well in a 96-well plate. Overall in 2D and 3D models, XenoB110-gfp-luc2 cells exhibited higher signals (about 20-fold) than Xeno284-gfp-luc2 cells. This could be due to a higher expression of luciferase in XenoB110-gfp-luc2 cells.

We then asked whether XenoLuc assay could be used to measure cell proliferation. Figure 1c shows that the luminescent signals increased in a time-dependent manner whereby the maximum growth of both xenograft cells was recorded at Day 6 and Day 4 for 2D and 3D culture models, respectively. Following that, the growth of both xenograft cells either reached plateau phase or declined. In another experiment, we captured the images of XenoB110-gfp-luc2 cells (2D and 3D cultures) under FITC channel using IN-Cell high content cellular analyser, and the GFP images were subjected to fluorescence intensity analysis by the IN-Cell Developer software. Additional file 2: Figure S2A and S2B show that the fluorescence intensity of non-depleted and mouse cell-depleted xenograft cells in 2D culture correlated positively to the number of cells seeded. The signal intensity also increased following the increase in cell proliferation from day 2 to 4. Similar correlations (between signal intensity and cell seeding numbers) were seen when XenoB110-gfp-luc2 cells were grown as 3D spheroids (Additional file 2: Figure S2C). However, in these 3D cultures, unlike luminescence signals, no obvious difference was found in fluorescence intensity between day-2 and day-4 readings.

### Characterization of XenoLuc assay

**Fig. 2:**
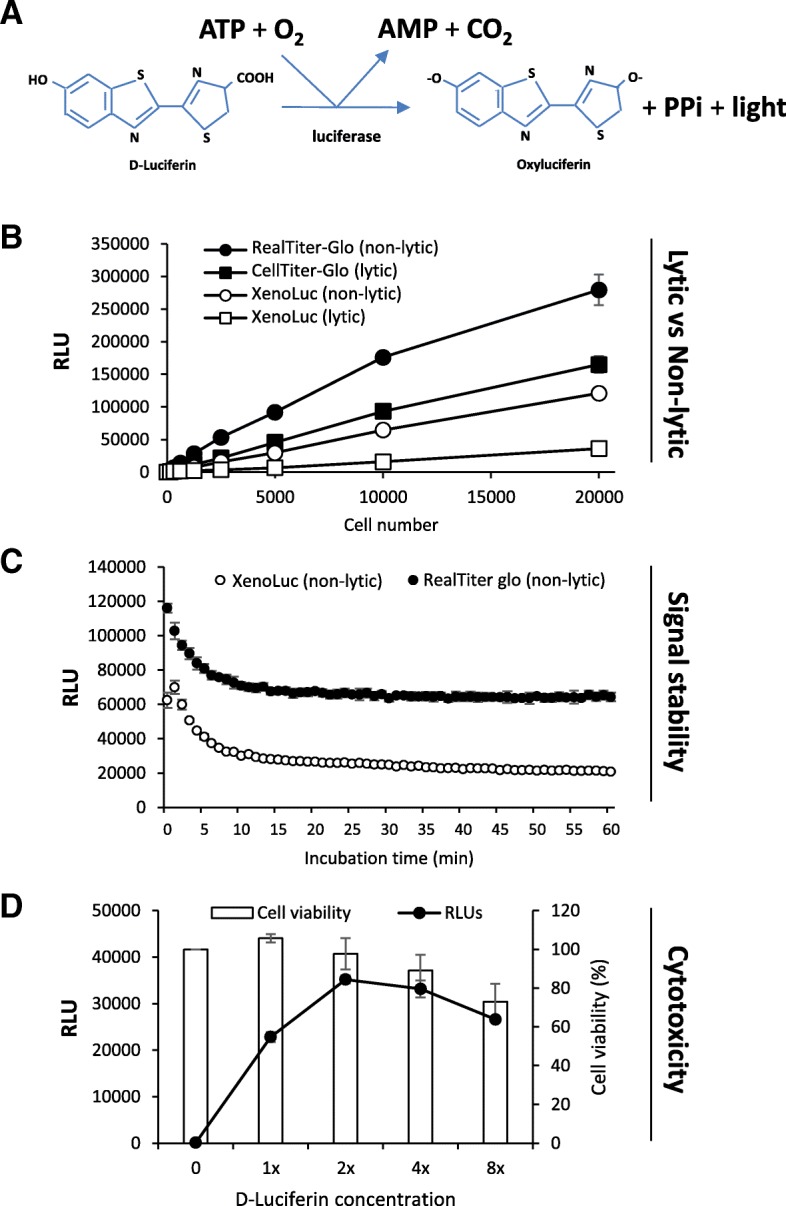
Characterization of XenoLuc assay. **a** Diagram showing the basic concept of luciferase/luciferin system. **b** Comparison of signal intensity detected by XenoLuc assay and two commercial assays: CellTiter-Glo (lytic) and RealTime-Glo (non-lytic). **c** Comparison of signal stability of XenoLuc and RealTime-Glo. **d** Cytotoxicity of D-Luciferin substrate after 24-h incubation as determined by MTS assay. Results are represented by average of triplicate from 3 mice ± SD

The principle of XenoLuc assay was illustrated in Fig. 2a. Light or luminescence is generated as the result of the enzymatic reaction between the D-Luciferin substrate and the intracellular luciferase in the presence of oxygen and adenosine triphosphate (ATP). In addition to the luminescence, other by-products are oxyluciferin, AMP, PPi (pyrophosphate), and carbon dioxide [9]. Figure 2b shows that the XenoLuc luminescent assay may be used either as endpoint (lytic) or non-destructive (non-lytic) assays. In line with the current trend of multiplexing cell-based assays, the non-lytic assay is preferable to the traditional endpoint assay because no cell lysis is required, and the same well may be multiplexed for various assays [10]. Comparatively, the luminescent signals detected by XenoLuc assay were relatively lower than that of commercial assays (Fig. 2b). RealTime-Glo and CellTiter-Glo employ exogenous luciferase and possibly more sensitive proprietary luciferin substrate along with other signal enhancers or co-factors such as Mg^2+^, recombinant ATP and Coenzyme A (CoA) [11]. The non-lytic version of XenoLuc assay was found to give higher signals than the lytic version of the assay at various tested cell numbers (Fig. 2b). We then assessed the luminescent signal stability of the XenoLuc assay in comparison with RealTime-Glo by recording the signals for 60 min at a 1-min interval. Figure 2c shows that the signals in both assays initially dropped from the first reading, and eventually stabilized after 10 min of incubation. These signals were stable for up to 60 min. We then selected 10 min as the incubation time for luciferin substrate with the luc2-expressing cells for subsequent experiments. As shown in Fig. 2d, the increase of D-luciferin substrate concentration to 2X from 1X increased the luminescence signal by 1.5 folds. The increment in signal declined when D-luciferin substrate was used at higher concentrations of 4X and 8X. More importantly, the presence of luciferin at 2X for 24 h did not cause toxicity to XenoB110-gfp-luc2 cells (Fig. 2d). This is consistent with Tiffen et.al [12]. However, cell viability was reduced to 89 and 73%, respectively when incubated with 4X and 8X luciferin substrate (Fig. 2 d). This explains the reduction in luminescent signals at these concentrations. Similar trends were also observed in Xeno284-gfp-luc2 cells (data not shown).

### XenoLuc assay for anti-cancer drug screening

**Fig. 3:**
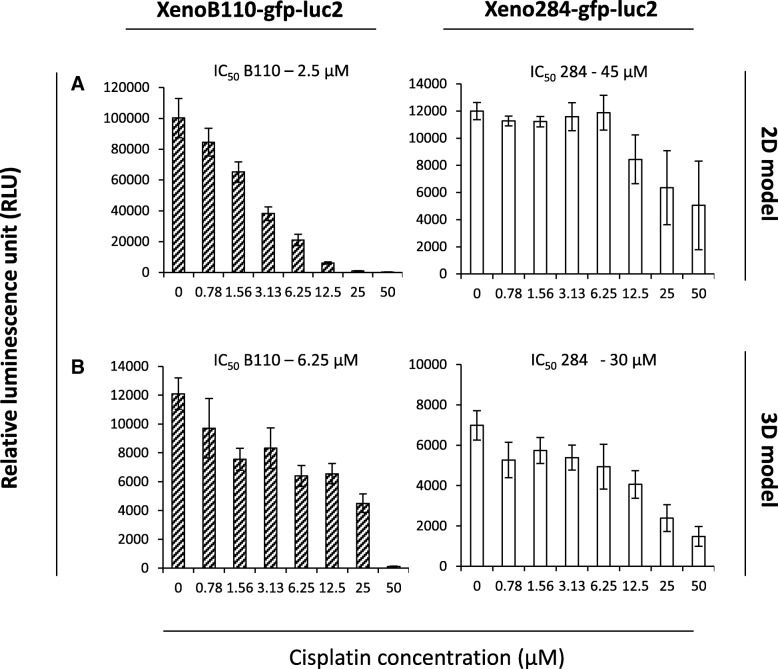
Inhibition of NPC xenograft cell viability by cisplatin as measured by XenoLuc assay. Xenograft cells (1 × 10^4^ cells/well) were seeded onto 96-well plates (***a***: 2D model; ***b***: 3D model) and treated with various concentrations of cisplatin as described in *Methods*. Luciferase activities were measured at Day 3. Results are represented by average of triplicate from 3 mice ± SD

Figure 3 shows that cisplatin exhibited dose-dependent cytotoxicity against both xenograft cells. Of the two xenograft cells, the decrease in cell viability of Xeno284-gfp-luc2 cells were less possibly because the cells were more resistant to cisplatin treatment (IC_50_ of 45 μM and 30 μM) as compared to XenoB110-gfp-luc2 cells (IC_50_ of 2.5 μM and 6.25 μM) in 2D and 3D culture models. A parallel comparison revealed that the overall luminescence of Xeno284-gfp-luc2 cells was lower than XenoB110-gfp-luc2 cells, consistent with Fig. 1**.** Nevertheless, comparisons were made possible because of the inclusion of untreated cells derived from the same cell isolates and carried out within the same experimental batch to avoid batch-to-batch variability.

### Determination of xenograft cell growth in a co-culture system

**Fig. 4:**
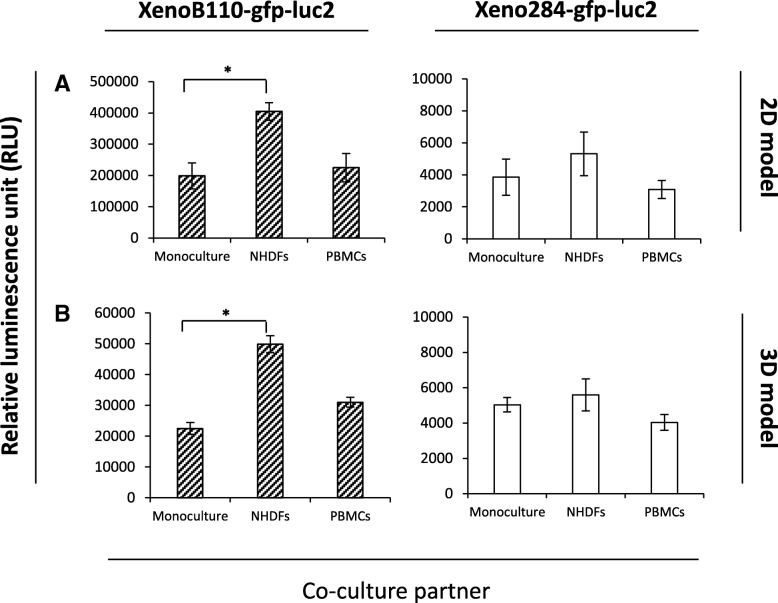
Measurement of luc2-modified xenograft cell growth in a co-culture system. Xenograft cells (1 × 10^4^ cells/well) were co-cultured with two different cell types as described in *Methods* in (**a**) 2D culture model; and (**b**) 3D culture model. Luminescence was measured at Day 4. Left panel: XenoB110-gfp-luc2; Right panel: Xeno284-gfp-luc2. Results are represented by average of triplicate from 3 mice ± SD. * *p* < 0.05

**Fig. 5:**
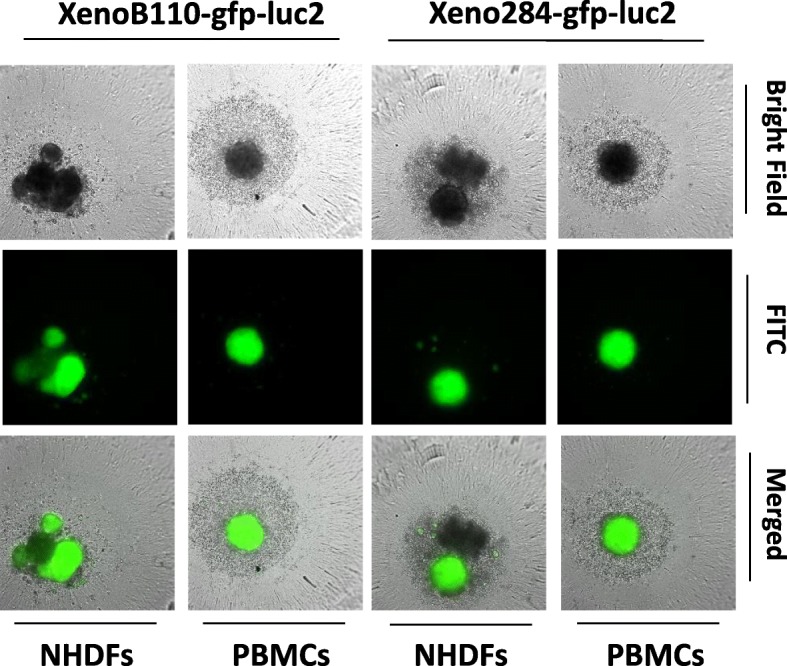
Microscopic examination of xenograft cells co-cultured with NHDFs and PBMCs in 3D culture model. 3D spheroids from xenograft cell-NHDF co-culture tended to form irregular shapes unlike those of xenograft cell-PBMC co-culture which were single, smooth and uniformed aggregates. (left panel: XenoB110-gfp-luc2; right panel: Xeno284-gfp-luc2)

As demonstrated by Fig. 2, XenoLuc assay specifically detected luc2-bearing tumour cells. Additionally, it would be beneficial if it could be used to measure the growth of tumour cells (either inhibition or enhancement) that were co-cultured with other cell types. To validate the use of XenoLuc assay in this application, we co-cultured the xenograft cells with either fibroblasts (NHDFs) (adherent cells) or PBMCs (suspension cells), and measured the luminescence signals to examine the growth inhibition or enhancement of xenograft cells. Figure 4a shows that the NHDFs enhanced xenograft cell growth in 2D culture model. However, only XenoB110-gfp-luc2 cells displayed a significant growth enhancement (*p* < 0.05). The growth enhancement in XenoB110-gfp-luc2 cells was also dependent on the number of seeded NHDFs (Additional file 3: Figure S3A). Co-culture of xenograft cells with PBMCs did not yield any significant difference in the growth as compared to that of monoculture (Fig. 4b and Additional file 3: Figure S3B). We then investigated whether this enhancing effect was reproducible in 3D culture model. Similarly, NHDFs significantly promoted XenoB110-gfp-luc2 cell growth in a seeding number-dependent manner (*p* < 0.05) (Fig. 4b and Additional file 3: Figure S3B). Figure 5 shows the microscopic analysis of GFP-labelled xenograft cells co-cultured with NHDFs and PBMCs individually in 3D models. Xenograft cell-NHDF co-cultures resulted in irregular and multiple spheroids compared to xenograft cell-PBMC co-cultures. Consistent with the luminescence data, the fluorescence intensity analysis showed that NHDFs promoted the growth of XenoB110-gfp-luc2 cells in both 2D and 3D cultures (Additional file 4: Figure S4).

## Discussion

In the present study, we developed a specific and sensitive luciferase-based assay known as XenoLuc to detect and measure real-time proliferation of luc2-transduced cells. The assay used freshly isolated xenograft cells which were passaged for not more than two times. This approach was established to circumvent the difficulty of establishing stable cell lines of NPC and allowed the use of xenografts which could not be propagated indefinitely as cell lines to be used for in vitro assays. This assay could be performed without lysing the cells, thereby allowing repeated and multiparametric measurements at multiple time-points in a single well (Fig. 1c). This assay could also capture the different growth patterns of culture models as shown in Fig. 1c. Both B110 and 284, the 3D culture models stopped growing at day 4, possibly due to the nutrient deprivation as basal RPMI1640 medium without any supplementation was used for culturing 3D spheroids. The luminescent signal was found to be stable up to 60 min, and D-Luciferin at working concentration was not toxic to the cells after 24-h exposure (Fig. 2c and d). We also showed that the assay is robust and could be applied on different cell model systems. Furthermore, it was reliably used for multiple applications, including drug/ inhibitor screening and growth measurement in a co-culture system (Figs. 3 and 4).

The luciferase/luciferin system has been adopted in various cell-based assays, and is widely used in biomedical research. This assay is sensitive, non-toxic, suitable for various applications and does not interfere with downstream applications [10, 13]. Conventional luciferase-based assays use exogenous or endogenous luciferase to generate bioluminescence upon the addition of an external substrate (luciferin). The amount of emitted light depends on the concentration of ATP, a widely accepted marker of viable cells that is also the limiting reactant in luciferase-based assays. Non-viable cells are unable to synthesize ATP and rapidly exhaust the existing ATPs through their endogenous ATPases. Hence, the amount of cellular ATP in viable cells determines the intensity of luminescent signal which is proportionate to the viable cell number [14, 15].

In addition to luminescence, GFP fluorescence of the transduced cells could also be detected, in contrast to the commercially available luminescence-based assays (Fig. 5 and Additional file 2: Figure S2C). The current assay utilizes dual *gfp* and *luc2* reporters to provide more cellular information. The endogenous luciferase (encoded by *luc2* reporter gene) in viable cells reacts chemically with an addition of luciferin to generate a luminescent signal, whereas the GFP signal (encoded by *gfp* reporter gene) provides a fluorescent visualization of transduced tumour cells. The 2-in-1 detection in XenoLuc assay helps researchers to differentiate cell types in a co-culture system. GFP signal intensity could also be determined using imaging software as a second measure of cell proliferation. It is noteworthy that traditional metabolic-based viability assays such as MTT, MTS and XTT do not discriminate the metabolic activity between cancer and stromal cells when they are cultured together. This results in an inability to measure accurately the viability of either cell population when the above metabolic assays are used in co-culture systems [16]. As luciferase and GFP expressions are confined within transduced cancer cells, measurement of luminescence and/or GFP fluorescence will accurately reflect cell proliferation changes in co-cultures. However, it should be noted that GFP fluorescence could not effectively measure the growth of 3D spheroid culture (Additional file 2: Figure S2C) unlike luminescence (Fig. 1c). This suggests that GFP fluorescence is less sensitive, possibly because the GFP signals do not penetrate well enough through the cells from within the 3D spheroids.

To demonstrate that XenoLuc assay is comparable, if not better than commercially available luminescent assays, we performed some of the experiments using our assay in parallel with CellTiter-Glo and RealTime-Glo kits from Promega, USA. Of note, the rate-limiting factor of both commercial assays is cellular ATP, while the supplied luciferin and luciferase are in excess. On the other hand, the limiting factor in XenoLuc could be the endogenous luciferase, cellular ATP, or both (Fig. 2a). By using these commercial kits as a benchmark, we showed that XenoLuc assay met several important criteria as a cell proliferation or viability assay, mainly being highly sensitive, rapid, non-toxic, quantitative, and yields stable signals (Fig. 2b–d). In addition, a comparison of the features among these assays was tabulated in Table 1. Despite having lower signal intensity, XenoLuc assay has several notable advantages over other luminescent assays which include having a lower cost per reaction but with its performance and robustness comparable to that of commercial assays. Thus, the XenoLuc assay appears to be the more cost-effective option for researchers. More importantly, XenoLuc assay may be used to measure specifically the proliferation of *gfp* and *luc2*-labeled cells of interest in multi-cellular systems such as co-cultures, unlike existing luminescent assays.

**Table 1:** Comparison of XenoLuc assay with commercial luminescent assays

Assay	Lytic/ Non-lytic	Price per reaction^a^	Incubation time	Signal intensity	Signal stability	Linearity (R^2^)
XenoLuc (In-house)	Lytic	RM 0.013 (USD 0.003)	15 min	36,133	> 1 h	0.9915
CellTiter-Glo (Promega, USA)	Lytic	RM 0.20 (USD 0.05)	1 h 15 min	165,000	> 1 h	0.9963
XenoLuc (In-house)	Non-lytic	RM 0.013 (USD 0.003)	10 min	120,960	> 1 h	0.999
RealTime-Glo (Promega, USA)	Non-lytic	RM 1.86 (USD 0.46)	1 h 15 min	279,400	> 1 h	0.9835

^a^Price acquired from Malaysia’s distributor

As a proof that XenoLuc assay could be a reliable tool for high-throughput screening for drug discovery, we tested the effect of cisplatin on the viability of our NPC xenograft cells. XenoLuc assay was able to show that although both transduced xenograft cells were sensitive to cisplatin, Xeno284-gfp-luc2 cells displayed some resistance to it as consistently observed in both 2D and 3D cultures. This observation could be attributed to the difference in disease type: Xeno-284 was established from recurrent NPC while XenoB110 was from untreated primary NPC. In the former, we speculate that the patient might have developed cisplatin resistance as a result of repetitive chemotherapy.

In the past decades, tumour cell monoculture has been used as a model for drug screening and the study of cancer biology. As increasing attention is drawn towards the complexity of tumour microenvironment, a co-culture model is believed to be more physiologically relevant for cancer research [2, 3]. The growth of XenoB110-gfp-luc2 cells was markedly enhanced when co-cultured with NHDFs (Fig. 4). In addition to luminescence data, it was also validated by the GFP fluorescence intensity analysis that the NHDFs promoted the growth of XenoB110-gfp-luc2 cells (Additional file 4: Figure S4). Unlike luminescence measurement that is in direct proportion to ATP levels [17], the fluorescence intensity analysis is not affected by the cellular ATP levels from the co-cultures. This growth enhancement was likely resulted from the activation of proliferative pathways upon direct cell-cell contact. NHDFs have been used as feeder cells to support cancer cell growth and to simulate epithelial-stromal cell interaction and paracrine signalling in an in vitro culture setting [18–21]. In addition, we also used PBMCs as the co-culture partner for xenograft cells. There were varying results in the growth of NPC xenograft cells when cultured in 2D and 3D models (Fig. 4). It could be due to the heterogeneity within PBMCs population as well as differential cell-cell contacts due to culture techniques.

## Conclusions

In conclusion, we have developed a specific, sensitive, real-time, non-toxic, and cost-effective luminescent assay to measure the proliferation of *luc2-*transduced cells. This robust assay known as XenoLuc assay could be used for multiple applications ranging from a simple experiment such as cell doubling time measurement, to complicated studies such as cell activation and proliferation or drug sensitivity/resistance screening in a co-culture system. This assay may serve as an important tool to specifically examine the role of a selected cell types in complex co-culture systems as well as for drug screening experiments.

## Methods

### Plasmid constructs and reagents

Lentiviral constructs (pMDL, RSV-REV, and VSVg) and gfp-luc2 DNA transfer plasmid were kindly provided by Marco Herold (Walter and Eliza Hall Institute of Medical Research, Melbourne, Australia). RPMI 1640 (#31800–022), fetal bovine serum (FBS) (#10082–139), GlutaMAX supplement (#35050–061), 0.05% Trypsin-EDTA (#25300–120), Pen-Strep (#15140–122), Pen-Strep-Fungizone (#15240–062), B-27 supplement (#17504–001), Insulin-Transferrin-Selenium (ITS) (#41400–045), Fibroblast growth factor (FGF)-Basic (bFGF) (#PHG0261), and Epidermal growth factor (EGF) (#PHG0311) were obtained from Gibco, USA. Hydrocortisone (#H0135), Collagenase Type II (#C6885), DPBS (#D5652), HEPES (#H3375), and Sodium Bicarbonate (#S5761) were purchased from Sigma-Aldrich, USA. DNase I (#90083) and Lipofectamine 2000 (#11668–027) were purchased from Thermo Fisher Scientific, USA. Collagenase/Dispase (#11097113001) was obtained from Roche, USA. Rho kinase (ROCK) inhibitor (#SCM075) and Polybrene (#TR-1003-G) were obtained from Merck Millipore, USA. XenoLight D-Luciferin substrate (#122799) was purchased from PerkinElmer, USA and stored in small aliquots at − 20 °C in the dark. CellTiter 96 AQueous One Solution Cell Proliferation Assay (MTS) (#G3580), CellTiter-Glo Luminescent Cell Viability Assay (#G7571), and RealTime-Glo MT Cell Viability Assay (#G9711) were from Promega, USA. Cisplatin (#15663–27-1) was purchased from Acros Organics, USA. RBC lysis solution was purchased from Qiagen, USA. All reagents were dissolved, stored, and used according to the manufacturer’s instruction.

### Cells and culture conditions

Primary normal human dermal fibroblasts (NHDFs) (#C-12300) were obtained from PromoCell, Germany and maintained using the medium and supplements supplied in the fibroblast growth medium kit (#C-23110) (PromoCell, Germany). HEK293T cells (#CRL-3216, ATCC, USA) were maintained in high glucose DMEM supplemented with 10% FBS (DMEM-10). Human peripheral blood mononuclear cells (PBMCs) were isolated from the blood samples collected from three donors via Ficoll-Paque (GE Healthcare, USA) centrifugation. All donors had agreed and signed the consent forms.

### Tissue dissociation and xenograft cell culture

Human NPC xenografts (XenoB110 and Xeno284) from NSG mice were processed as previously described by our group [8, 22]. All experiments were performed in accordance with the protocols approved by Animal Care and Use Committee (ACUC), Ministry of Health, Malaysia. Briefly, mice were humanely euthanized using carbon dioxide gas (flow rate of between 1.3 to 3.8 L/min) or cervical dislocation by trained personnel using protocols approved by the Animal Care and Use Committee. Harvested xenograft tumours were transferred to cold DPBS supplemented with 1X Pen-Strep-Fungizone, and processed immediately. Surrounding blood capillaries, fat, and/or necrotic tissue were removed and washed twice with DPBS with 1X Pen-Strep-Fungizone. The tumours were minced into 2 to 4-mm fragments and incubated with the appropriate dissociation solution (2 mg/mL Collagenase Type II and 200 U/mL DNase I for XenoB110; 1 mg/mL Collagenase/Dispase for Xeno284) on a belly dancer shaker with a constant agitation for 1–2 h at 37 °C, 5% CO_2_. This was followed with an addition of complete growth media (RPMI-10, 1X Pen-Strep-Fungizone, 1X B-27 Supplement, 1X ITS, 10 μM ROCK Inhibitor, 10 ng/mL EGF, and 10 ng/mL bFGF), after which the entire suspension was filtered through 40 μm nylon mesh cell strainer (BD Falcon, USA). The released cells were centrifuged at 800 rpm for 5 min. RBC lysis solution was added onto the cells prior to centrifugation at 800 rpm for 5 min. The cells were then processed with the mouse cell depletion kit (MACS Miltenyi Biotec #130–104-694) following the manufacturer’s instruction to enrich for human NPC xenograft cells. The enriched cells were cultured in the above mentioned complete growth medium for 2D monolayer culture, whereas basal RPMI-10 was used for 3D spheroid culture. The cells were detached using 0.05% Trypsin-EDTA at 70–80% confluence and were sub-cultured at 1:3 dilutions. To our best experience, xenograft cells could be maintained up to passage four (P4) in vitro in monolayer culture, and spheroids could be formed by medium exchange and using a commercial spheroid plate. To minimize biological variations, we only used freshly isolated xenograft cells which were passaged for up to 2 times for experiments. We used the terms xenograft cells for these cells in contrast to xenograft tumours in mice.

### Lentivirus production and cell transduction

1 × 10^6^ HEK293T cells were seeded on 10-cm culture dishes and incubated for 24 h. The gfp-luc2 lentiviral transfer vector with packaging and envelope plasmids (pMDL, RSV-REV and VSVg) were combined at a ratio of 4:2:1:1, respectively, and mixed with Lipofectamine 2000 for transfection according to the manufacturer’s protocol. The viral supernatant was collected at 24, 48 and 72 h post-transfection. Cellular debris was pelleted by centrifugation at 1500 rpm for 5 min at 4 °C followed by filtration using PVDF Millex-HV filter, 0.45 μm (Millipore #SLHV033RS). The filtered lentivirus supernatant was then concentrated using Lenti-X Concentrator (Clontech #PT4421–2) according to the manufacturer’s instruction. For cell transduction, the mouse cell-depleted xenograft cells were seeded on a 10-cm culture dish and transduced with the concentrated lentivirus at MOI 2.0 in the presence of 10 μg/mL Polybrene for 24 h. GFP positive cells were selected using a FACSAria SORP (BD Biosciences, USA).

### XenoLuc assay

XenoLuc assay was performed as follows for both 2D and 3D culture experiments. At each time point, 2X D-Luciferin substrate diluted in RPMI-10 was dispensed into each cell-containing well at 1:1 ratio. The plate was gently agitated and incubated at room temperature in the dark for 10 min to stabilize the luminescence. The luminescent signal of each plate was then read by EnVision multi-label plate reader (PerkinElmer) using the ultrasensitive mode. Following the completion of a time-point reading, the D-Luciferin containing medium was removed from the well. The cells were washed two times gently with 200 μL RPMI-10, and then replenished with 100 μL of fresh complete media until the subsequent reading. Alternatively, images of GFP-expressing xenograft cells from each well were captured using IN Cell Analyzer 2000, and the GFP fluorescence intensity was measured and compared using the IN Cell Developer software. For the lytic-based format of XenoLuc assay, the protocol described by Oba and co-workers was modified and adapted [23]. Briefly, PBS supplemented with 1% Triton X-100 and 1X protease inhibitor cocktail (Millipore #539134) was used as cell lysis buffer and the lysis was performed for 10 min. After lysis, the assay buffer containing 5 mM MgCl_2_, 100 mM Tris-HCl (pH 7.8) and 2X D-Luciferin substrate was added to the well to generate the luminescence. The plate was immediately measured after 15-min incubation at room temperature in the dark.

### CellTiter-Glo and RealTime-Glo

CellTiter-Glo (end-point assay) and RealTime-Glo (continuous assay) were performed according to the manufacturer’s instructions. To examine if the XenoLight D-Luciferin substrate reagent was toxic to the xenograft cells, cell viability assay (CellTiter 96 Aqueous One) was performed on xenograft cells which had been exposed to the substrate for 24 h.

### Applications of XenoLuc assay

We evaluated the suitability of the assay for inhibitor/drug screening and co-culture studies using both 2D and 3D culture models. For the drug screening study, cisplatin was used as the standard drug. Xenograft cells were similarly plated in 2D and 3D culture conditions as described above. Cells were then treated with various concentrations of cisplatin (0, 0.78, 1.56, 3.13, 6.25, 12.5, 25, and 50 μM) for 72 h followed by the XenoLuc assay. Prior to co-culturing, both NHDFs and PBMCs were gamma-irradiated separately at 35 Gy. For both 2D and 3D cultures, 10,000 xenograft cells were seeded simultaneously with the irradiated co-culture partner cells at 1:1 ratio into the same well. After an incubation of 4 days, XenoLuc assay was performed to measure the proliferation of xenograft cells in the presence of either irradiated NHDFs or PBMCs.

### Statistical analysis

For each experiment using xenograft cells, three tumours were harvested from three separate mice, and each resulting xenograft cells were seeded into three wells per isolate of xenograft cells for subsequent experiments. Each data point was represented by average of triplicate from 3 mice ± SD. GraphPad Prism 6 software (GraphPad Software Inc.) was used to calculate the IC_50_ value of cisplatin against xenograft cells. R^2^ value was also determined to show the linear association between two parameters in a linear curve. SPSS version 22.0 software (IBM SPSS, USA) was used for analysis of statistical significance. One-way analysis of variance with Bonferroni correction and Student’s *t* test were used to determine if the differences between two different groups are significant. The results presented are mean + standard deviations. *p* < 0.05 was considered statistically significant.

## Additional files

Additional file 1: **Figure S1.** Relative luminescence units (RLUs) signals of non-adhering B110 xenografts cells. Luciferase activities were measured 30 min after cell seeding (day-0 reading). (A) XenoB110-gfp-luc2; (B) Xeno284-gfp-luc2. (PPTX 41 kb)

Additional file 2: **Figure S2.** Cell proliferation analysis of XenoB110-gfp-luc2 by GFP fluorescence intensity using IN-CELL Developer software. (A) Non-depleted xenograft cells; (B) Mouse cell-depleted xenograft cells; (C) 3D spheroids. The images inset shows an increasing size of spheroids captured at day 4 proportionate to cell seeding number. R^2^ shows the linear correlation of luminescence and cell number. (PPTX 62 kb)

Additional file 3: **Figure S3.** Evaluation of xenograft cell growth by co-culture in 2D and 3D models. Xenograft cells (1 × 10^4^ cells/well) were co-cultured with various cell number of NHDFs and PBMCs in both (A) 2D; and (B) 3D model. Left panel: XenoB110-gfp-luc2; Right panel: Xeno284-gfp-luc2. (PPTX 44 kb)

Additional file 4: **Figure S4.** Analysis of XenoB110-gfp-luc2 cell growth in a co-culture system by GFP fluorescence intensity using IN-CELL Developer software. XenoB110-gfp-luc2 cells (1 × 10^4^ cells/well) were co-cultured with two different cell types as described in *Methods* in (A) 2D culture model; and (B) 3D culture model. GFP fluorescence intensity was determined at Day 4. (PPTX 38 kb)

## Data Availability

The datasets used and/or analysed during the current study are available from the corresponding author on reasonable request.

## Abbreviations

2D: Two-dimensional
3D: Three-dimensional
ATP: adenosine triphosphate
EBER: Epstein-Barr virus-encoded small RNA
EBV: Epstein-Barr virus
ECM: extracellular matrix
EGF: epidermal growth factor
FACS: fluorescence-activated cell sorting
FGF: fibroblast growth factor
GFP: green fluorescence protein
H&E: Haematoxylin and Eosin
ITS: Insulin-Transferrin-Selenium
LCA: Leukocyte common antigen
luc: luciferase
NHDFs: normal human dermal fibroblasts
NPC: nasopharyngeal carcinoma
NSG: NOD *scid* gamma
PAN-CK: Cytokeratin Pan
PBMC: peripheral blood mononuclear cell
PBS: phosphate buffered saline
PDTX: patient-derived tumour xenograft
RBC: red blood cells
RFU: relative fluorescence unit
RLU: relative luminescence unit
ROCK: Rho kinase
