# Supplementary figures and images for: Development of a luciferase/luciferin cell proliferation (XenoLuc) assay for real-time measurements of Gfp-Luc2-modified cells in a co-culture system
Source: BMC Biotechnol. 2019 Jun 14;19:34. doi: 10.1186/s12896-019-0528-4 (PMC6570829; doi:10.1186/s12896-019-0528-4)

## Slide 1
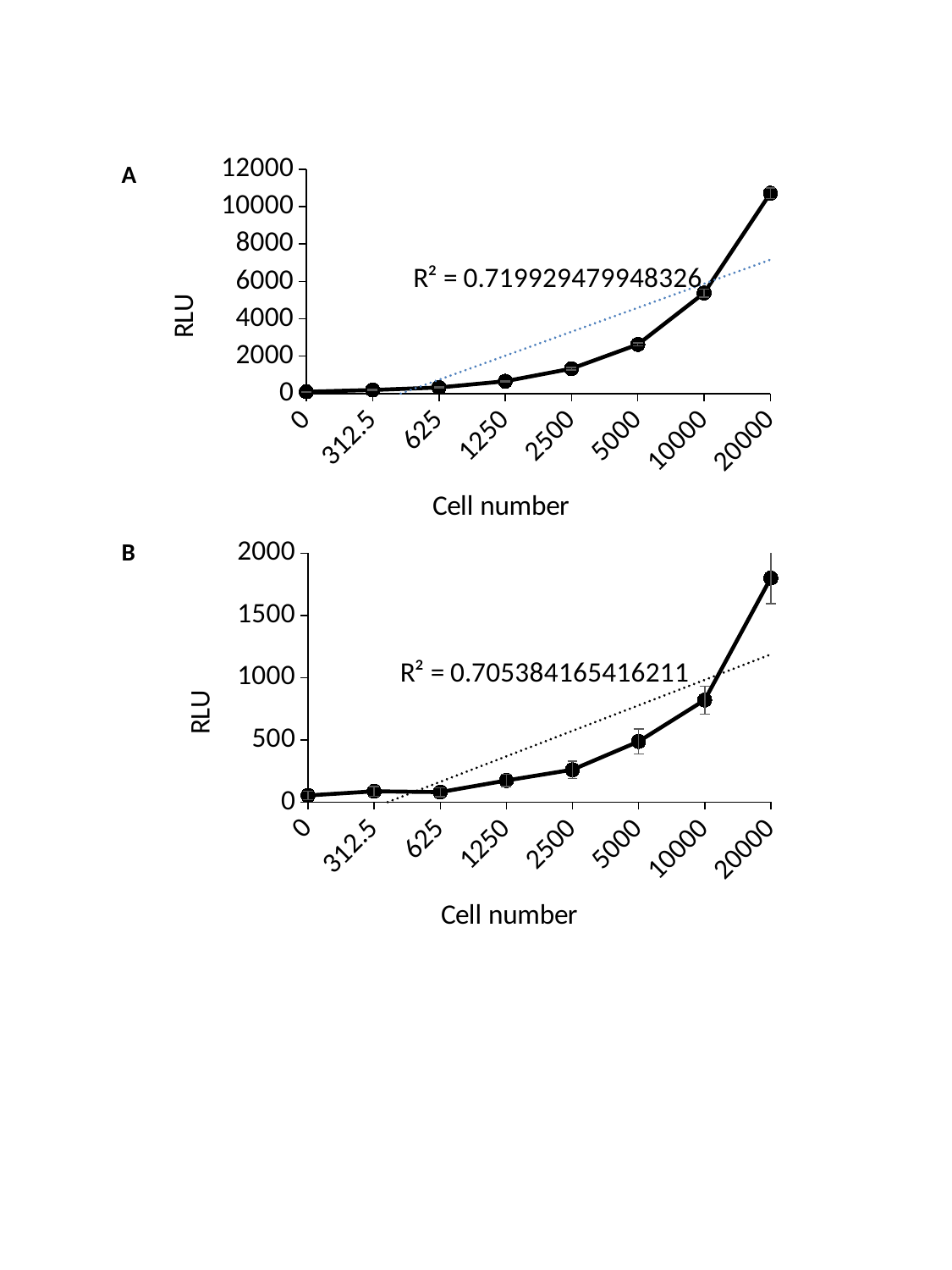

### Chart
| Category | |
|---|---|
| 0 | 98.3333333333333 |
| 312.5 | 190.833333333333 |
| 625 | 325.833333333333 |
| 1250 | 660.0 |
| 2500 | 1333.33333333333 |
| 5000 | 2628.33333333333 |
| 10000 | 5380.0 |
| 20000 | 10717.5 |A
B
### Chart
| Category | |
|---|---|
| 0 | 53.3333333333333 |
| 312.5 | 86.6666666666667 |
| 625 | 80.0 |
| 1250 | 173.333333333333 |
| 2500 | 260.0 |
| 5000 | 486.666666666667 |
| 10000 | 820.0 |
| 20000 | 1800.0 |

Supplement: Supplementary file 1 — Figure S1. Relative luminescence units (RLUs) signals of non-adhering B110 xenografts cells. Luciferase activities were measured 30 min after cell seeding (day-0 reading). (A) XenoB110-gfp-luc2; (B) Xeno284-gfp-luc2. (PPTX 41 kb) [file 12896_2019_528_MOESM1_ESM.pptx]
