# Supplementary material for: Development of a luciferase/luciferin cell proliferation (XenoLuc) assay for real-time measurements of Gfp-Luc2-modified cells in a co-culture system
Source: BMC Biotechnol. 2019 Jun 14;19:34. doi: 10.1186/s12896-019-0528-4 (PMC6570829; doi:10.1186/s12896-019-0528-4)
Supplement: Supplementary file 2 — Figure S2. Cell proliferation analysis of XenoB110-gfp-luc2 by GFP fluorescence intensity using IN-CELL Developer software. (A) Non-depleted xenograft cells; (B) Mouse cell-depleted xenograft cells; (C) 3D spheroids. The images inset shows an increasing size of spheroids captured at day 4 proportionate to cell seeding number. R2 shows the linear correlation of luminescence and cell number. (PPTX 62 kb) [file 12896_2019_528_MOESM2_ESM.pptx]

## Slide 1
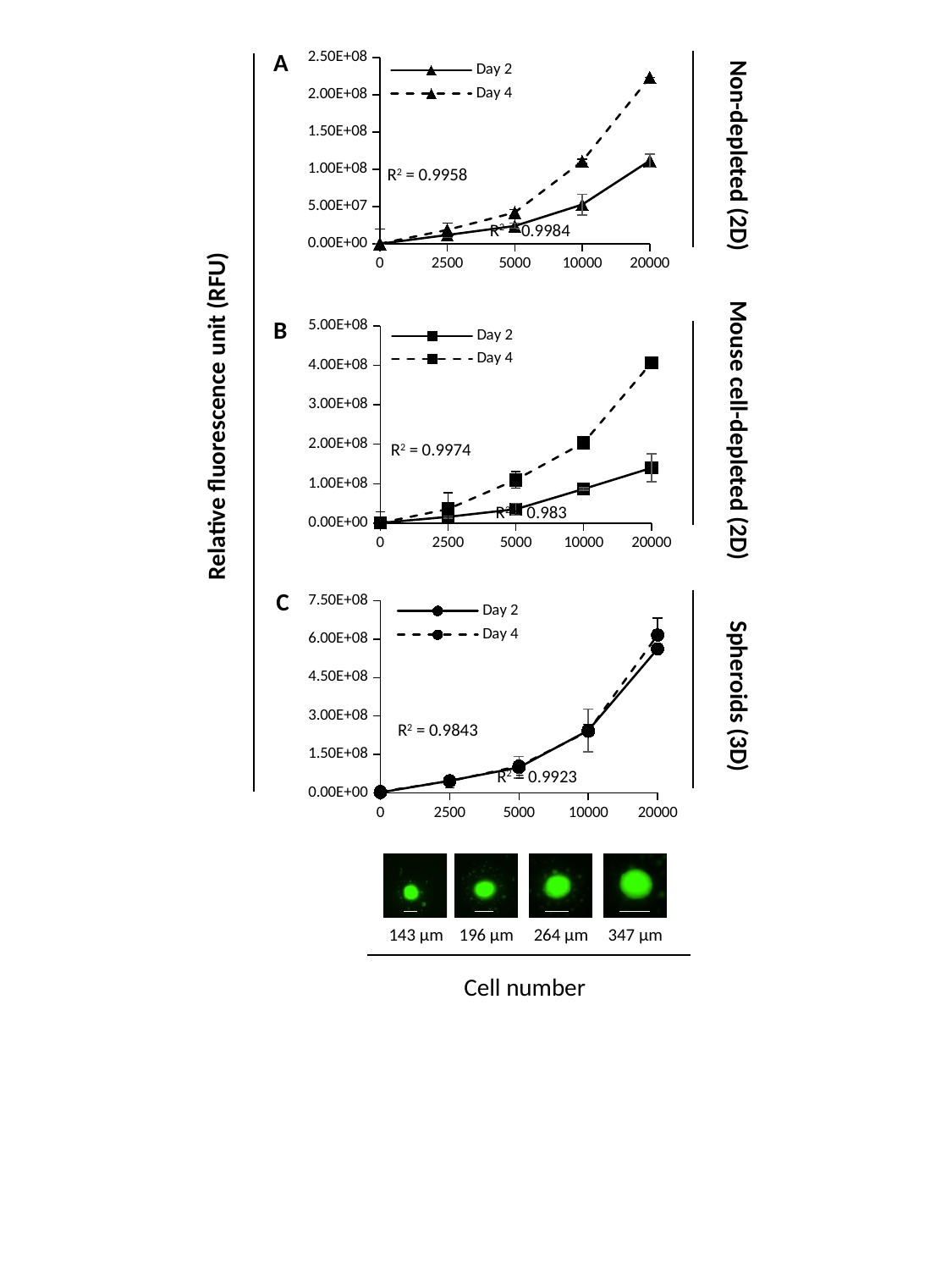

### Chart
| Category | Day 2 | Day 4 |
|---|---|---|
| 0 | 100000.0 | 235232.0 |
| 2500 | 11912785.0 | 18766666.6666667 |
| 5000 | 23962066.3333334 | 42083333.3333333 |
| 10000 | 52663404.6666667 | 110833333.333333 |
| 20000 | 111655026.833333 | 223166666.666667 |A
Non-depleted (2D) Mouse cell-depleted (2D) Spheroids (3D)
R2 = 0.9958
R2 = 0.9984
Relative fluorescence unit (RFU)
### Chart
| Category | Day 2 | Day 4 |
|---|---|---|
| 0 | 532612.0 | 496016.333333335 |
| 2500 | 16009148.3333333 | 35733333.3333333 |
| 5000 | 35404513.0 | 109833333.333333 |
| 10000 | 86756196.6666667 | 203833333.333333 |
| 20000 | 140013333.333333 | 406666666.666667 |B
R2 = 0.9974
R2 = 0.983
C
### Chart
| Category | Day 2 | Day 4 |
|---|---|---|
| 0 | 1231523.0 | 4119833.2 |
| 2500 | 46905026.0 | 45492382.0 |
| 5000 | 98953618.0 | 104153618.0 |
| 10000 | 243663333.333333 | 240263333.0 |
| 20000 | 562176666.666666 | 616435333.2 |R2 = 0.9843
R2 = 0.9923
 143 µm 196 µm 264 µm 347 µm
Cell number
