# Supplementary material for: Development of a luciferase/luciferin cell proliferation (XenoLuc) assay for real-time measurements of Gfp-Luc2-modified cells in a co-culture system
Source: BMC Biotechnol. 2019 Jun 14;19:34. doi: 10.1186/s12896-019-0528-4 (PMC6570829; doi:10.1186/s12896-019-0528-4)
Supplement: Supplementary file 3 — Figure S3. Evaluation of xenograft cell growth by co-culture in 2D and 3D models. Xenograft cells (1 × 10^4^ cells/well) were co-cultured with various cell number of NHDFs and PBMCs in both (A) 2D; and (B) 3D model. Left panel: XenoB110-gfp-luc2; Right panel: Xeno284-gfp-luc2. (PPTX 44 kb) [file 12896_2019_528_MOESM3_ESM.pptx]

## Slide 1
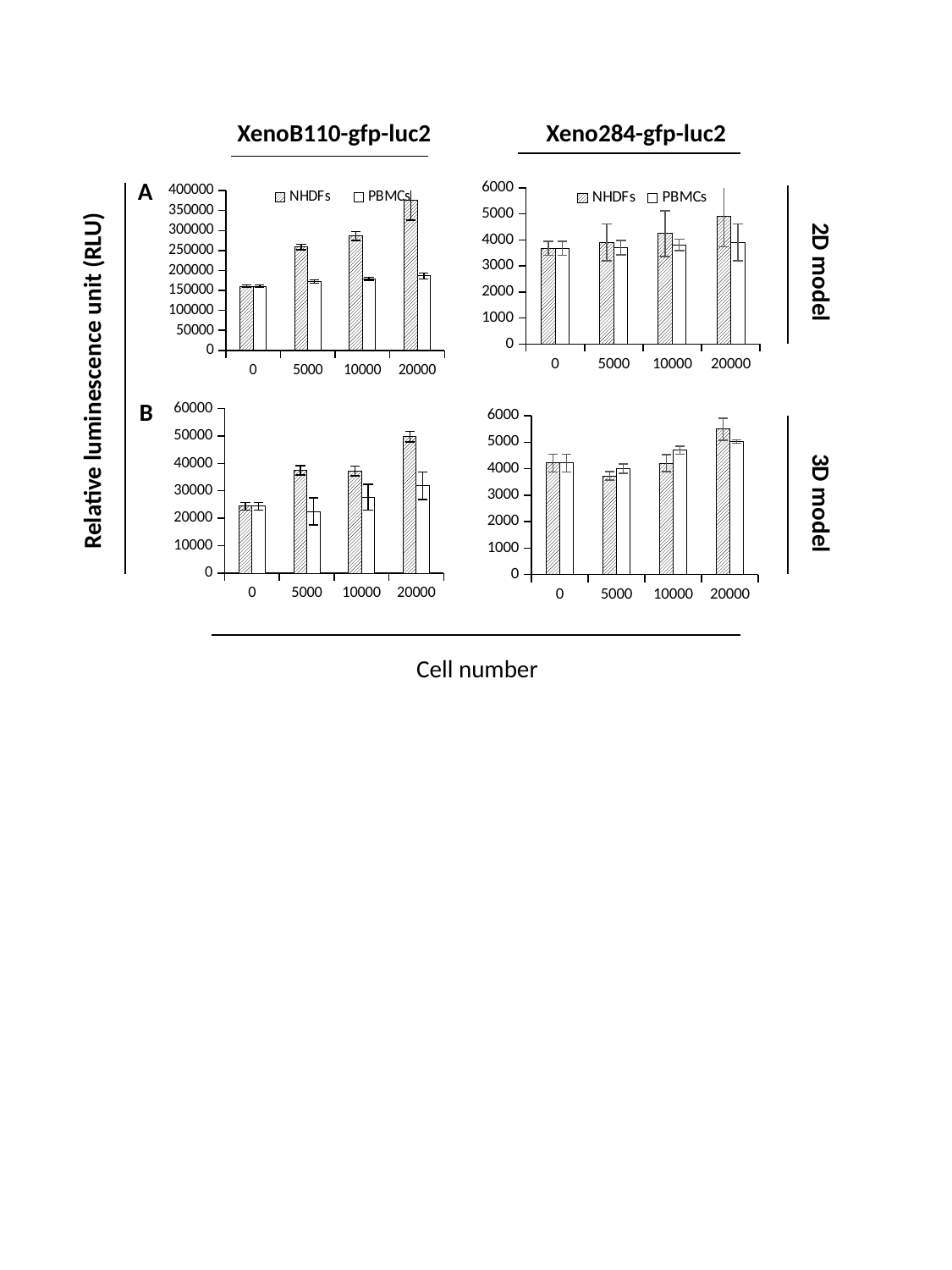

XenoB110-gfp-luc2
Xeno284-gfp-luc2
### Chart
| Category | NHDFs | PBMCs |
|---|---|---|
| 0 | 3680.0 | 3680.0 |
| 5000 | 3906.66666666665 | 3706.66666666665 |
| 10000 | 4243.33333333333 | 3810.0 |
| 20000 | 4906.66666666673 | 3906.66666666665 |
### Chart
| Category | NHDFs | PBMCs |
|---|---|---|
| 0 | 160453.333333333 | 160453.333333333 |
| 5000 | 258520.0 | 172373.333333333 |
| 10000 | 286146.666666667 | 179186.666666667 |
| 20000 | 375720.0 | 186266.666666667 |A
 2D model 3D model
Relative luminescence unit (RLU)
### Chart
| Category | NHDFs | PBMCs |
|---|---|---|
| 0 | 4213.33333333333 | 4213.33333333333 |
| 5000 | 3730.0 | 4006.66666666665 |
| 10000 | 4206.66666666673 | 4696.66666666673 |
| 20000 | 5490.0 | 5026.66666666673 |
### Chart
| Category | NHDFs | PBMCs |
|---|---|---|
| 0 | 24386.6666666667 | 24386.6666666667 |
| 5000 | 37506.6666666666 | 22480.0 |
| 10000 | 37253.3333333333 | 27640.0 |
| 20000 | 49746.6666666666 | 31786.6666666667 |B
Cell number
