# Supplementary material for: Development of a luciferase/luciferin cell proliferation (XenoLuc) assay for real-time measurements of Gfp-Luc2-modified cells in a co-culture system
Source: BMC Biotechnol. 2019 Jun 14;19:34. doi: 10.1186/s12896-019-0528-4 (PMC6570829; doi:10.1186/s12896-019-0528-4)
Supplement: Supplementary file 4 — Figure S4. Analysis of XenoB110-gfp-luc2 cell growth in a co-culture system by GFP fluorescence intensity using IN-CELL Developer software. XenoB110-gfp-luc2 cells (1 × 10^4^ cells/well) were co-cultured with two different cell types as described in Methods in (A) 2D culture model; and (B) 3D culture model. GFP fluorescence intensity was determined at Day 4. (PPTX 38 kb) [file 12896_2019_528_MOESM4_ESM.pptx]

## Slide 1
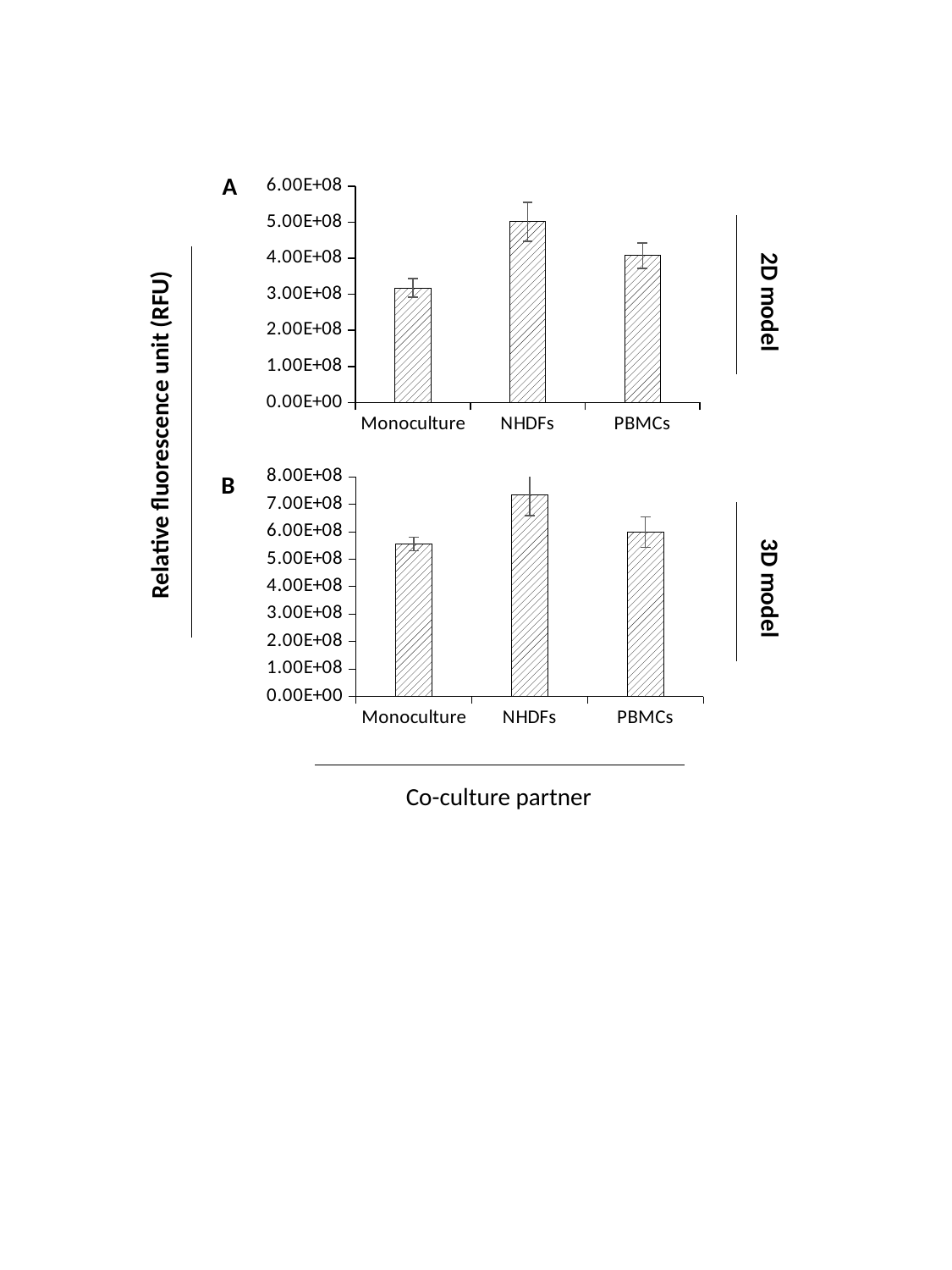

A
### Chart
| Category | |
|---|---|
| Monoculture | 317985833.3333333 |
| NHDFs | 501435555.5555556 |
| PBMCs | 407547777.7777778 | 2D model 3D model
Relative fluorescence unit (RFU)
B
### Chart
| Category | |
|---|---|
| Monoculture | 555154444.4444444 |
| NHDFs | 735243333.3333334 |
| PBMCs | 598484444.4444444 |Co-culture partner
